# Research progress on ferroptosis and PARP inhibitors in ovarian cancer: action mechanisms and resistance mechanisms

**DOI:** 10.3389/fphar.2025.1598279

**Published:** 2025-04-24

**Authors:** Jiqing Zhang, Dan Ouyang, Mu Liu, Yuting Xiang, Zhongjun Li

**Affiliations:** ^1^ The First Clinical Medical College, Guangdong Medical University, Zhanjiang, China; ^2^ Department of Obstetrics and Gynecology, Dongguan People’s Hospital, Dongguan, China

**Keywords:** ovarian cancer, PARP inhibitor, ferroptosis, action mechanism, resistance mechanism, synthetic lethality

## Abstract

Ovarian cancer, a gynecologic malignancy with high mortality rates, faces persistent therapeutic challenges due to acquired resistance and frequent recurrence with conventional therapies. While poly (ADP-ribose) polymerase (PARP) inhibitors have primarily transformed clinical outcomes through the synthetic lethality mechanism, their long-term efficacy remains constrained by therapeutic resistance. Ferroptosis, a novel programmed cell death modality characterized by iron-dependent lipid peroxidation, has emerged as a promising therapeutic frontier in oncology. This review is the first to summarize the mechanisms of action and resistance associated with both ferroptosis and PARP inhibitors in ovarian cancer.

## 1 Introduction

Epidemiological data indicate ovarian cancer accounted for approximately 313,959 new cases and 207,252 deaths globally in 2020 (Sung et al., 2021). Notably, 70% of ovarian cancer diagnoses occur at advanced stages, principally due to the lack of disease-specific symptoms and validated screening methods during early pathogenesis (Kehoe et al., 2015). Conventional treatment for advanced-stage disease has traditionally relied on cytoreductive (tumor debulking) surgery followed by platinum-based chemotherapy. Despite these interventions, clinical data reveal that 70% of epithelial ovarian cancer patients receiving optimal debulking surgery followed by first-line chemotherapy subsequently experience disease recurrence (Kuroki and Guntupalli, 2020).

Ovarian cancer treatment has undergone significant paradigm shifts through clinical integration of poly (ADP-ribose) polymerase (PARP) inhibitors, a breakthrough class of precision therapeutics. This evolution has established six PARP inhibitors in the current treatment landscape: olaparib, niraparib, fluzoparib, rucaparib, talazoparib, and pamiparib, reflecting structural evolution from first-to third-generation compounds (Ni et al., 2021; Chu et al., 2022). The most prevalent genomic damage occurs as single-strand break, whereas double-strand break represent the more cytotoxic DNA lesions. PARP plays a critical role in detecting and repairing chromosomal damage induced by single-strand break, with pharmacological inhibition resulting in the accumulation of DNA fragmentation that exacerbates genomic instability (Gupte et al., 2017). In contrast, endogenous DNA repair systems primarily utilize homologous recombination to resolve double-strand break. Loss-of-function mutations in breast cancer susceptibility genes (BRCA1/2) or other homologous recombination repair genes frequently lead to homologous recombination deficiency (Chiappa et al., 2021). Therapeutically, PARP inhibitors selectively inhibit single-strand break repair processes in homologous recombination deficient cancers, thereby promoting the accumulation of excessive double-strand break that ultimately culminates in selective tumor cell death through the principle of synthetic lethality (Kim et al., 2021). Nevertheless, with expanding clinical adoption of PARP inhibitors in first-line maintenance regimens for ovarian cancer, the emergence of acquired resistance has become a critical clinical barrier requiring urgent resolution.

The year 2012 witnessed a pivotal discovery in cell death biology when Dixon et al. originally conceptualized ferroptosis: an iron-dependent regulated cell death pathway distinguished by three defining characteristics—iron dysregulation, uncontrolled lipid peroxidation, and reactive oxygen species (ROS) overaccumulation (Dixon et al., 2012). In contrast to canonical cell death pathways (apoptosis, necrosis, autophagy), ferroptosis is defined by unique biochemical and morphological signatures (Hirschhorn and Stockwell, 2019). Converging translational research evidence has established the pathophysiological significance of this oxidative death mechanism in ovarian carcinogenesis and treatment resistance (Chen B. et al., 2023; Guo et al., 2024; Kapper et al., 2024). Despite PARP inhibitors becoming therapeutic mainstays in ovarian cancer, their mechanistic intersections with ferroptotic pathways remain poorly characterized. To our knowledge, this is the first comprehensive review to systematically delineate the interface between PARP inhibitors and ferroptosis in ovarian cancer, encompassing both action mechanisms and resistance mechanisms. No prior review has specifically focused on this emerging therapeutic synergy, underscoring the novelty and clinical relevance of our work. The purpose of this review is to elucidate the latest mechanistic advancements in ferroptosis and PARP inhibitors within ovarian cancer.

## 2 Overview of ferroptosis

To better contextualize the mechanisms associated with ferroptosis and PARP inhibitors in ovarian cancer, this section outlines the biochemical foundations of ferroptosis and its distinct features compared with classical cell death pathways. Iron, as the fourth-most abundant element in terrestrial crust, acts as an essential cofactor in human physiology by regulating oxygen transport systems and facilitating biogenic processes including DNA replication, ATP synthesis, and protein biosynthesis. Ferroptosis—a unique iron-dependent cell death mechanism driven by lipid peroxidation—differs fundamentally from apoptosis, necrosis, or autophagy. Functionally, ferroptosis demonstrates pathognomonic ultrastructural features: cytoplasmic volume reduction, mitochondrial membrane densification with cristae remodeling or loss, and outer mitochondrial membrane rupture, while maintaining intact nuclear architecture during execution (Mou et al., 2019). At the molecular level, ferroptosis hinges on three interconnected events: glutathione depletion, functional inactivation of glutathione peroxidase 4 (GPX4, a key enzyme that detoxifies lipid peroxides), and iron/ROS overload (Pan et al., 2022). Ferrous iron potently potentiates non-enzymatic peroxidation of polyunsaturated fatty acid containing phospholipids through Fenton chemistry. Ferroptosis originates from metabolic rewiring and catastrophic homeostatic collapse of the ROS generation-scavenging equilibrium (Pratt et al., 2011). The key metabolic mechanisms are driven by four core mechanisms: abnormal iron metabolism, lipid ROS generation, dysregulation of the antioxidant system, and accumulation of lipid peroxides (Jiang et al., 2021). Ferroptosis has been implicated in the pathogenesis and clinical progression of multiple pathological conditions, spanning cancers, neurodegenerative diseases, ischemia-reperfusion injury, and acute kidney injury (Li et al., 2021; Ryan et al., 2023; Pourhabib Mamaghani et al., 2025; Yu et al., 2025). Emerging evidence underscores the critical role of ferroptosis in ovarian cancer, particularly in therapeutics, prompting intensive investigation into its potential mechanistic interplay with PARP inhibitors.

Recent advances in ferroptosis research are redefining pharmacological frameworks for PARP inhibitors, uncovering previously unrecognized mechanistic intersections and driving innovative therapeutic strategies. Low-dose olaparib (50 mg/kg) mitigates sepsis-induced myocardial dysfunction with preserved cardiac function and boosts survival outcomes through dual mechanisms involving enhanced mitophagy promoting clearance of damaged mitochondria and transcriptional repression of key ferroptosis regulators, effectively reducing lipid peroxides and iron accumulation (Liu et al., 2024). PARP mutation is one of the mechanisms of PARP inhibitor resistance. The novel PARP1 degrader NN3 circumvents PARP inhibitor resistance by selectively inducing ferroptosis in p53-positive (wild-type p53 or missense mutant p53) breast cancer cells through downregulating the solute carrier family 7 member 11 (SLC7A11) pathway (Li et al., 2022). Furthermore, NN3 exhibits minimal toxicity and robust activity *in vivo*. Niraparib potentiates radiotherapy responses by augmenting cytosolic double-stranded DNA accumulation, which activates cyclic GMP-AMP synthase signaling to drive activating transcription factor 3-mediated SLC7A11 suppression in colorectal cancer, while concomitantly enhancing CD8^+^ T cell immunosurveillance (Shen et al., 2022). Systems biology-driven development of ferroptosis prognostic signatures in acute myeloid leukemia reveals that combination therapy with talazoparib and erastin (a ferroptosis inducer) synergistically inhibits leukemic stem cell clonogenic potential and metastatic competence (Wu et al., 2023). This multimodal action establishes PARP inhibitor-ferroptosis crosstalk as a therapeutic vulnerability, with PARP inhibitors serving dual regulatory roles in both mediating treatment efficacy and overcoming resistance mechanisms. In addition, pharmacological synergy with adjuvant agents significantly enhances therapeutic effects across multiple diseases. Given the critical role of ferroptosis in cancer cell death, recent studies have explored its interaction with PARP inhibitors in ovarian cancer.

## 3 Action mechanisms of ferroptosis and PARP inhibitors

Having established the core mechanisms of ferroptosis, we now examine its interplay with PARP inhibitors in ovarian cancer, focusing on two key themes: “synthetic lethality” and drug synergy.

### 3.1 Ferroptosis and “synthetic lethality”

**FIGURE 1:**
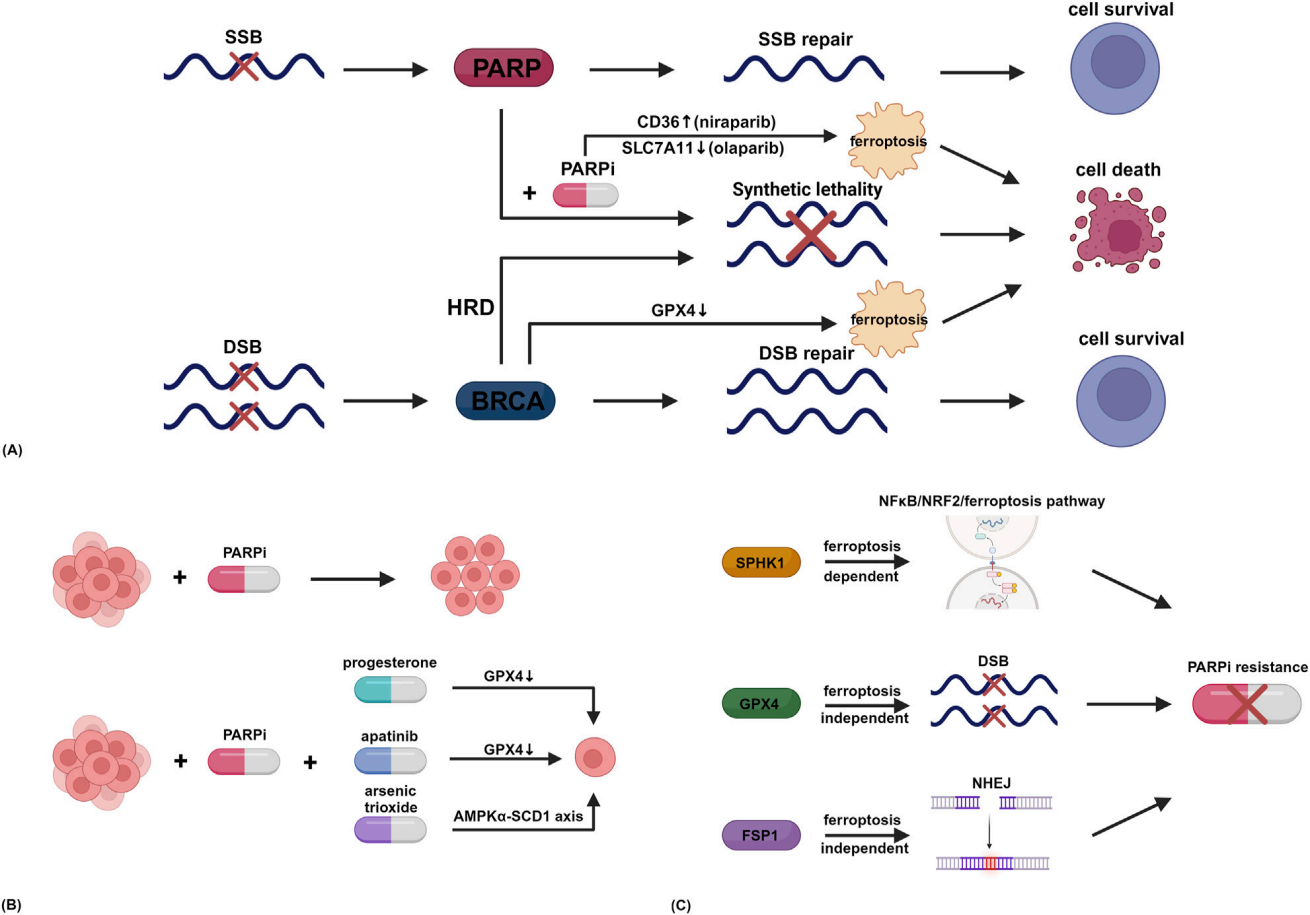
**(A)** Ferroptosis and “synthetic lethality”. **(B)** Ferroptosis and drug synergy. **(C)** Resistance mechanisms. SSB, single-strand break; DSB, double-strand break; PARP, poly (ADP-ribose) polymerase; BRCA, breast cancer susceptibility gene; PARPi, PARP inhibitor; HRD, homologous recombination deficiency; SLC7A11, solute carrier family 7 member 11; GPX4, glutathione peroxidase 4; SCD1, stearoyl-coenzyme A desaturase 1; NRF2, nuclear factor erythroid 2-related factor 2; SPHK1, sphingosine kinase 1; FSP1, ferroptosis suppressor protein 1; NHEJ, non-homologous end joining. Created in https://BioRender.com.

Synthetic lethality, a foundational concept in PARP inhibitor therapy, refers to the phenomenon where simultaneous disruption of two genes (e.g., PARP and BRCA) leads to cell death, whereas individual disruptions do not. This principle underpins the clinical efficacy of PARP inhibitors in homologous recombination deficient cancers. Recent studies reveal that synthetic lethality may intersect with ferroptosis, offering expanded therapeutic opportunities. Niraparib induces dysregulated fatty acid uptake and lipid accumulation in ovarian cancer cells through transcriptional upregulation of the fatty acid transporter CD36, triggering ferroptosis and consequently suppressing peritoneal metastasis (Jin et al., 2025). Notably, this therapeutic effect is independent of the status of p53 and BRCA, with preclinical models demonstrating that genetic ablation of CD36 or pharmacological inhibition of ferroptosis significantly attenuates antitumor responses. This study reveals a new mechanism through which niraparib targets lipid metabolism in the tumor microenvironment via ferroptosis, providing theoretical support for its clinical application. Olaparib downregulates SLC7A11 in a p53-dependent manner, impairing glutathione biosynthesis and leading to ferroptosis through lipid peroxidation (Hong et al., 2021). The combination of ferroptosis inducers enhances the antitumor effect of olaparib in BRCA wild-type ovarian cancer. BRCA1 catalyzes lysine 6-linked ubiquitination-mediated proteasomal degradation of GPX4, thereby sensitizing cancer cells to ferroptosis (Xie et al., 2024). This non-canonical tumor-suppressive function expands the role of BRCA1 beyond DNA damage repair. BRCA1 deficiency results in GPX4 accumulation, conferring ferroptosis resistance and facilitating ovarian cancer progression. Therapeutic synergy emerges from combined GPX4 inhibitor and PARP inhibitor treatment, which cooperatively induce ferroptosis in BRCA1-deficient ovarian cancer cells and potently inhibit tumor growth. This innovative therapeutic paradigm transcends traditional DNA repair-centric approaches, offering novel combinatorial strategies for BRCA1-deficient cancers. While PARP inhibitors and BRCA1 are established core components of synthetic lethality, the studies above demonstrate their ability to mediate antitumor effects in ovarian cancer through a novel ferroptosis-dependent pathway that transcends the traditional confines of synthetic lethality interactions Figure 1. (A) Summarizes the above information on ferroptosis and “synthetic lethality”.

### 3.2 Ferroptosis and drug synergy

While synthetic lethality remains a cornerstone of PARP inhibitor therapy, clinical resistance necessitates combinatorial approaches. Ferroptosis, with its unique reliance on lipid peroxidation and iron metabolism, provides a mechanistic basis for synergistic drug interactions. This section explores how ferroptosis induction amplifies PARP inhibitor efficacy across diverse molecular contexts, including BRCA wild-type and platinum-resistant ovarian cancer. Progesterone enhances ferroptosis by upregulating palmitoleic acid biosynthesis, thereby significantly improving niraparib efficacy in ovarian cancer cells regardless of BRCA mutation status, with a more pronounced effect observed in wild-type BRCA cells (Wu et al., 2024). Progesterone activates stearoyl-coenzyme A desaturase 1 (SCD1) to promote lipid peroxidation, inhibits GPX4 activity, triggers mitochondrial dysfunction and ROS accumulation, and synergizes with niraparib to induce DNA damage and ferroptosis. Clinical data from this study demonstrate that patients with high progesterone receptor expression and low GPX4 levels exhibit prolonged survival when treated with progesterone-niraparib combination therapy, underscoring ferroptosis as a key mediator of PARP inhibitor therapeutic synergy. In p53 wild-type ovarian cancer cells, the anti-angiogenic agent apatinib synergizes with olaparib to downregulate GPX4 through dual suppression of nuclear factor erythroid 2-related factor 2 (NRF2) and autophagy pathways, eliciting ferroptosis (Yue et al., 2023). Conversely, p53-mutant cells exhibit NRF2 resistance, which can be reversed by p53 reactivation. NRF2 activation or autophagy induction abrogates this ferroptotic vulnerability, providing a translational rationale for p53 status-guided precision combination therapy. Olaparib and arsenic trioxide demonstrate synergistic efficacy in platinum-resistant ovarian cancer cells through dual mechanisms: augmented DNA damage (γ-H2AX and cleaved caspase-3 upregulation) inducing apoptosis, and AMPKα-SCD1 axis-mediated lipid metabolic rewiring that promotes lethal lipid peroxide accumulation, thereby inducing ferroptosis (Tang et al., 2022). Collectively, these findings establish ferroptosis as a pivotal mechanism underlying PARP inhibitor combination strategies and provide innovative therapeutic paradigms for overcoming platinum resistance in ovarian cancer Figure 1. (B) Summarizes the above information on ferroptosis and drug synergy.

Platinum sensitivity serves as a functional biomarker guiding PARP inhibitor clinical application, as PARP inhibitor monotherapy demonstrates limited efficacy in platinum-resistant ovarian cancer (Audeh et al., 2010; Fong et al., 2010; Tew et al., 2020). However, emerging evidence reveals that PARP inhibitors achieve significant therapeutic benefits in platinum-resistant disease through ferroptosis-mediated pharmacological synergy. This oxidative cell death pathway not only enhances the antitumor activity of PARP inhibitors but also expands their clinical applicability. While progesterone’s novel ferroptosis-inducing mechanism was first identified in ovarian cancer, the ferroptotic mechanisms of apatinib and arsenic trioxide were previously established in other diseases (Tian et al., 2021; Zhao et al., 2021; Feng et al., 2022; Wang et al., 2022; Chen J. et al., 2023; Jin et al., 2024; Su et al., 2024). Furthermore, multiple pharmacological agents exert therapeutic effects through ferroptosis modulation in a wide variety of disorders, including artemisinin, metformin, simvastatin, sulfasalazine, and sorafenib (Yang et al., 2024; Zhao et al., 2024; Deng et al., 2025; Wang et al., 2025; Wu et al., 2025). These drugs hold significant potential when used in conjunction with PARP inhibitors to treat ovarian cancer.

## 4 Resistance mechanisms of ferroptosis and PARP inhibitors

Despite therapeutic advances, resistance remains a critical barrier. This review categorizes resistance mechanisms into two distinct classes: ferroptosis-dependent and non-ferroptotic pathways.

### 4.1 Resistance mechanism associated with ferroptosis

Ferroptosis evasion often underlies resistance to PARP inhibitors. Sphingosine kinase 1 (SPHK1) drives NRF2 transcription and suppresses ferroptosis via NF-κB p65 activation, thereby conferring resistance to olaparib in ovarian cancer cells (Teng et al., 2025). Inhibition of SPHK1 attenuates this pathway, reduces NRF2 expression, and induces lethal lipid peroxidation and ferroptosis, ultimately reversing olaparib resistance. Preclinical validation demonstrates that SPHK1 inhibition synergizes with olaparib to suppress tumor growth, establishing ferroptosis modulation as a promising therapeutic strategy to overcome resistance to PARP inhibitors.

### 4.2 Resistance mechanisms unrelated to ferroptosis

While ferroptosis suppression is a well-characterized resistance mechanism, recent studies reveal unexpected roles for canonical ferroptosis regulators (e.g., GPX4, FSP1) in driving resistance through DNA repair rewiring. By eliminating lipid peroxides, GPX4 preserves cellular oxidative equilibrium and serves as a crucial regulator of ferroptosis (Forcina and Dixon, 2019). In homologous recombination proficient ovarian cancer cells, GPX4 inhibition significantly elevates intracellular ROS levels, subsequently inducing oxidative stress-mediated DNA double-strand break (Gu et al., 2024). This ROS-mediated DNA damage potently sensitizes homologous recombination proficient ovarian cancer cells to olaparib and niraparib. The study demonstrates that combining GPX4 inhibitor with PARP inhibitor elicits synergistic efficacy in homologous recombination proficient ovarian cancer cells, distinguishing this mechanism from the ferroptosis pathway. Ferroptosis suppressor protein 1 (FSP1) functions as a key anti-ferroptotic factor independent of GPX4, suppressing lipid peroxidation via ubiquinone reduction (Mao et al., 2021; Mishima et al., 2022). A study reveals that FSP1 orchestrates PARP inhibitor resistance in BRCA-proficient ovarian cancer cells through a non-ferroptotic pathway (Miao et al., 2024). Specifically, FSP1 interacts with Ku70 to catalyze its poly (ADP-ribosyl)ation (PARylation), facilitating the recruitment of DNA-dependent protein kinase catalytic subunit (DNA-PKcs) to double-strand break sites and activating non-homologous end joining repair. Consequently, FSP1 inhibition disrupts Ku70 PARylation, impairing DNA-PKcs complex formation, leading to non-homologous end joining repair defects and accumulation of DNA damage, thereby reversing PARP inhibitor resistance.

GPX4 and FSP1 are central regulators of ferroptosis. However, resistance mechanisms associated with PARP inhibitors have unexpectedly been found to operate independently of ferroptosis in ovarian cancer. This observation highlights the need to further explore non-ferroptotic pathways linked to these ferroptosis-related proteins in the future Figure 1. (C) Summarizes the above information on resistance mechanism.

## 5 Summary and outlook

Ovarian cancer remains a critical threat to women’s health, where PARP inhibitors have emerged as transformative therapies, yet their clinical utility is constrained by persistent resistance challenges. This review delineates the mechanisms of action and resistance associated with ferroptosis and PARP inhibitors in ovarian cancer. Traditionally, PARP inhibitors exert antitumor effects through synthetic lethality in DNA repair-deficient tumors. However, recent studies reveal lipid metabolic reprogramming (CD36-mediated lipid accumulation) and redox homeostasis disruption (BRCA1-dependent ubiquitination degradation of GPX4) linked to PARP inhibitors and ferroptosis, extending beyond the DNA repair-centric framework. These findings provide new insights into PARP inhibitor efficacy and expand the theoretical boundaries of synthetic lethality. By demonstrating the synergistic effects of PARP inhibitors with other drugs, this review proposes highly effective combination regimens for ovarian cancer. At the same time, the potential value of combining several listed drugs (artemisinin, metformin, simvastatin, sulfasalazine, and sorafenib) with PARP inhibitors is highlighted, significantly broadening clinical application scenarios. This review not only identifies the SPHK1-NF-κB-NRF2 axis (associated with ferroptosis) as a driver of PARP inhibitor resistance by inhibiting ferroptosis, but also uncovers the resistance of non-classical DNA repair pathway (unrelated to ferroptosis) mediated by FSP1-Ku70, proposing the “ferroptosis dependent and independent” resistance mechanism. It provides a multi-target intervention strategies for overcoming clinical PARP inhibitor resistance. In conclusion, a robust association exists between ferroptosis and PARP inhibitors in ovarian cancer.

Current research on ferroptosis and PARP inhibitors remains largely confined to preclinical investigations (*in vitro* and animal models), with limited clinical translation to date. Future directions should prioritize the identification of validated biomarkers to optimize synergistic therapeutic precision while systematically evaluating combination regimen toxicity profiles and corresponding mitigation strategies. Given PARP inhibitors’ expanding applications across malignancies, the mechanisms of interaction between PARP inhibitors and ferroptosis warrant exploration beyond ovarian cancer. Computational modeling of drug response predictors (e.g., machine learning-based algorithms) could accelerate preclinical-to-clinical translation by forecasting optimal drug combinations and toxicity thresholds.

The emerging convergence between ferroptosis and PARP inhibitor development holds transformative potential to bridge mechanistic discoveries with clinical implementation. This review establishes a novel mechanism-to-clinical translational framework that promises to redefine precision medicine paradigms for ovarian cancer while providing innovative therapeutic blueprints to overcome chemoresistance in solid malignancies. Future studies should prioritize the clinical feasibility of mechanism-driven therapeutic strategies and ultimately achieve a closed-loop continuum spanning theoretical innovation, technological breakthroughs, and patient benefit.
